# Assessing the Time to Ward Transfer in Patients Presenting to the Emergency Department with an Acute Hip Fracture: A Closed-loop Audit

**DOI:** 10.7759/cureus.6794

**Published:** 2020-01-28

**Authors:** Marc C Grant-Freemantle, Robert M Kenyon, John Gibbons, Sean O Flynn, Martin Davey, Neil Burke

**Affiliations:** 1 Trauma and Orthopaedics, Beaumont Hospital, Dublin, IRL; 2 Trauma and Orthopaedics, Royal College of Surgeons, Dublin, IRL

**Keywords:** hip fracture, boa blue book standards, transfer to ward, trauma, emergency

## Abstract

Introduction

The British Orthopaedic Association and British Geriatric Association Blue Book guidelines for patients presenting acutely with a hip fracture stipulate that the patient should be admitted to an acute orthopedic ward within four hours of presentation to the emergency department (ED).

Materials and methods

A retrospective review of all patients who presented to the ED with a hip fracture diagnosed on plain film X-Ray over an eight-week period by a single auditor. Time of arrival, time to X-ray, time of blood draw, time to orthopedic referral, time to orthopedic review, and time to arrival at the orthopedic ward were documented. A policy change stipulating that orthopedics on call would prospectively review potential hip fracture patients prior to definitive workup was initiated. The same parameters were re-audited following this intervention over a six-week period.

Results

Pre-intervention, the mean time to orthopedic review was 83 minutes with a mean time to ward of 417 minutes. Post-intervention, the mean time to orthopedic review was 76 minutes with a mean time to ward of 333 minutes. When orthopedic trainees were on call, the mean time to review was 37.5 minutes with a mean time to ward of 294 minutes.

Conclusions

While we were able to demonstrate an improvement in orthopedic response times, this did not significantly improve time to ward transfer. This highlights a number of other areas that need to be optimized to improve compliance with best practice guidelines.

## Introduction

Hip fractures are a major burden on public health systems and remain a considerable driver of morbidity and mortality in geriatric populations. In Ireland, in 2018, 3,751 patients sustained a hip fracture. This equated to 70,231 acute bed days in Irish hospitals, with a mean stay of 18.7 days, costing the health service an estimated 45 million euro in acute care alone [1].

Hip fractures are often referred to as ‘fragility fractures,’ which reflects the typical patient who sustains them [2]. In 2018, the mean age of a hip fracture patient was 81 years old and 25% of these patients had a degree of cognitive impairment. The majority of these patients suffer from osteopenia or osteoporosis and multiple medical comorbidities, making these challenging patients to manage perioperatively with prolonged rehabilitation needs [1-2]. Up to 20% of patients will not regain their pre-hip fracture functional status, meaning that following a hip fracture, many patients have increased care needs, increasing the demand on families and community supports [2]. Furthermore, data from the United Kingdom (UK) indicate a high degree of mortality in these patients, with a 10% mortality at 30 days and a 30% mortality at one year post-fracture [3].

In recognition of the increasing frailty and complexity of these patients, standards were introduced in the UK for optimal hip fracture care. The key performance indicators (KPIs) outlined in clinical guidelines in acute hip fracture care have moved away from the traditional surgical metrics of morbidity and mortality and, instead, focus on the timely delivery of high-quality multidisciplinary care [1,4]. The most widely cited of these guidelines is the jointly issued British Orthopaedic Association (BOA) and British Geriatric Association (BGA) Blue Book Standards, which outline the key standards of care for hip fracture patients (as given in the table in the Appendix) [5].

A key factor in ensuring the delivery of this high-quality care has been the introduction of a best practice tariff (BPT) associated with the above KPIs. The BPT is a payment to hospitals operating on hip fracture patients aged 60 years or older who meet these KPIs [1]. The introduction of the BPT has been shown in UK studies to reduce time to theater from admission, reduce mortality at 30 days, and reduce mortality at one year in patients who met BPT criteria [3,6].

Overcrowding in Irish emergency departments (EDs) remains a significant and growing problem, as patient flow from the ED to the ward remains slow due to ongoing bed shortages [7]. Subsequently, it is unsurprising that BOA guideline one, which stipulates that patients should be admitted to an orthopedic ward within four hours of presentation, is the most poorly complied with of the BOA guidelines. The national compliance rate in Ireland was 17% in 2018 and 11% in 2017. Our institution had a compliance rate in 2018 of just 11% [1]. It has been shown that centers that have a hip fracture alert system are more likely to have a higher compliance rate with BOA guideline one. Despite our institution having such an alert system in place, the authors felt that engagement with the system was low. It was believed that the manner in which the alert system was being employed and the subsequent response from clinicians was an area that could be targeted to improve upon the efficiency and quality of the hip fracture pathway. Given the evidence that BPT drives changes in practice with measurable, positive impacts on patient care, as well as the potential for improvement with the hip fracture alert system we wanted to assess our institutions' performance in meeting guideline 1. In addition, we sought to identify key time points along the patient journey through the ED and assess if we could improve times to ward transfer by pre-emptively assessing suspected hip fracture patients at the earliest possible opportunity prior to a definitive referral by the ED.

## Materials and methods

A retrospective review of hip fracture patients who presented to our tertiary referral center’s ED over an eight-week period between July 13, 2019, and September 14, 2019, was performed. Patients were identified based on the orthopedic department’s theater log and their medical charts were reviewed. The time and date that the patient arrived in the ED were extracted from the ED triage sheet and was designated as time zero. The time of orthopedic referral was extracted from the ED doctor’s notes. For instances in which this was not documented, the time of referral was determined from the orthopedic senior house officer (SHO) notes, as well as the time of clinical review of patient and subsequent time of admission of the patient. If these events were not documented in medical notes, nursing notes were reviewed for the timestamps of the review of respective doctors. If this information was not available, this time was deemed not to have been recorded. The time of arrival on the ward was extracted from the nursing admission notes.

The online picture archiving and communication system (PACS) was reviewed so that the timings of plain film pelvic X-rays could be documented. The information extracted from this system was the time the X-ray was ordered, the time the radiographer sent porters to collect the patient for X-ray, and the time that the X-ray was filmed.

The online internal blood result system was also reviewed to determine the time when blood was drawn from the patient. For all patients, the time that the full blood count (FBC) was drawn was used as a marker for phlebotomy time.

To be included in the analysis, patients had to have been diagnosed with a hip fracture on plain film X-rays at the time of index admission in the ED. Patients who sustained a hip fracture within the hospital had a delayed diagnosis following presentation or when there was a requirement for higher-order imaging to make a definitive diagnosis were excluded from the analysis.

The intervention introduced as part of this audit was to provide all SHOs who provided orthopedic on-call cover in the institution with a protocol requesting the specific and precise documentation of times of hip fracture alert, a referral from ED, and time of orthopedic review and admission. The institution had previously operated a generic hip fracture alert system, which was activated in triage if a suspected hip fracture presented to the ED, alerting the on-call SHO and orthopedic trauma ward. It was requested that the orthopedic on-call team review any prospective hip fracture patients following this generic alert and prior to a definitive referral from the ED. This was then reinforced by the on-call orthopedic registrar where possible.

Following the introduction of this intervention, the audit process, as outlined above, was continued for a further six weeks until November 01, 2019. All anonymized data was stored in an encrypted Excel file (Microsoft Corporation, Redmond, Washington) and time in minutes from time zero (ED arrival) to listed timestamps was recorded (Table 1).

**Table 1:** Recorded timestamps

Timestamps
Time from arrival in emergency department to blood received
Time from arrival in emergency department to X-ray ordered
Time from X-ray ordered to arrived
Time from X-ray arrived to filmed
Arrival in emergency department to filmed X-ray
Time from arrival in emergency department to orthopedic referral
Time from orthopedic referral to orthopedic review
Time from orthopedic admission to orthopedic ward
Time from arrival in emergency department to orthopedic ward

The mean time between each of these timestamps was calculated in the pre- and postintervention group, as well as subgroup analysis for night admissions (20:00 to 08:00) and when an orthopedic team SHO was on call for orthopedics.

Statistical analysis was performed using STATA version 16 (Stata Corp, College Station, Texas). An unpaired t-test was used to assess the differences between the pre- and postintervention groups. A p-value of <0.05 was considered statistically significant.

## Results

A total of 71 patients met the inclusion criteria. Medical charts were available for 65 of these patients. Four out of 47 charts were unavailable from the pre-intervention group and two out of 24 in the postintervention group.

Table 2 outlines the mean time in minutes between each key endpoint in the pre- and postintervention groups.

**Table 2:** Mean time in minutes for key endpoints in patients presenting with an acute hip fracture to the emergency department X 8 out of 47 referrals without a completed AP pelvis X-ray (17%); * 3 out of 24 referrals without a completed AP pelvis X-ray (12.5%); xx 8 out of 47 referrals without blood (17%); ** 6 out of 24 referrals without blood (25%) AP: anteroposterior

	Time blood sent from arrival in emergency department	Time to ordered X-ray from emergency department arrival	Time from X-ray ordered to arrived	Time to filmed X-ray from arrival	Time to completed X-ray from arrival in emergency department	Time to orthopedic referral from admission	Time to orthopedic review from referral	Time from admission by orthopedics to ward	Time to ward from arrival in emergency department
Mean time pre-intervention (mins)	88^xx^	73	27	42	145	143^x^	83	209	417
Mean time postintervention (mins)	119**	29	29	33	92	85*	76	210	333
Mean time total population (mins)	98	59	28	39	128	125	78	209.5	388

The mean time difference for each of these timestamps between the pre- and postintervention groups, as well as the statistical significance for these time differences, are outlined in Table 3.

**Table 3:** Mean time difference for timestamps between the pre- and postintervention groups and associated p-values

	Time blood sent from arrival in emergency department	Time to ordered X-ray from emergency department arrival	Time from X-ray ordered to arrived	Time to filmed X-ray from arrival	Time to completed X-ray from arrival in emergency department	Time to orthopedic referral from admission	Time to orthopedic review from referral	Time from admission by orthopedic to ward	Time to banks from arrival in emergency department
Mean time difference in postintervention group	31-minute increase	44-minute decrease	2-minute increase	9-minute decrease	53-minute decrease	58-minute decrease	7-minute decrease	1-minute increase	84-minute decrease
p-value (time difference between groups)	0.755	0.007	0.591	0.238	0.238	0.020	0.484	0.501	0.126

A total of 23 patients (32.3% of acute hip fracture admissions) presented at night (20:00 to 08:00). The timings for this subgroup are outlined in Table 4. There was no statistically significant difference in time to ward transfer in the population of patients who presented at night as compared to those who presented during the day (p = 0.342).

**Table 4:** Mean timestamps for total population of patients presenting at night *14 out of 23 referrals without blood (60.8%); X 6 out of 23 referrals without a completed AP pelvis X-ray (26%) AP: anteroposterior

	Time blood sent from arrival in emergency department	Time to ordered X-ray from emergency department arrival	Time from X-ray ordered to arrived	Time to filmed X-ray from arrival	Time to completed X-ray from arrival in emergency department	Time to orthopedics referral from admission	Time to orthopedics review from referral	Time from admission by orthopedics to ward	Time to banks from arrival in emergency department
Mean time total population (night) (mins)	134*	70	27	36	134	142^X^	65	258	419

Eleven percent of acute hip fracture admissions were when an SHO who worked on the orthopedic team was on call for orthopedics. The timings for this subgroup are outlined in Table 5. There were no statistically significant differences in review times (p = 0.902) or ward transfer times (p = 0.955) in patients who were admitted by an orthopedic team SHO as opposed to a general SHO covering orthopedics.

**Table 5:** Mean timestamps for when an orthopedic team SHO is on call for orthopedics SHO: senior house officer

	Time to ortho review from referral	Time from admission by orthopedic to ward	Time to ward from arrival in emergency department
Mean time total population (orthopedic senior house officer admission) (mins)	37.5	177	294

The overall documentation rate of key parameters was also reviewed and is outlined in Table 6.

**Table 6:** Documentation rate of key parameters

	Time admitted to ward	Time of ortho referral	Time of ortho review	Time of hip fracture alert
Documentation rate pre-intervention	68.09%	70.30%	93.60%	10.60%
Documentation rate postintervention	73.92%	65.3	74%	26%
Documentation rate total population	70.50%	69%	88.80%	15.50%

The overall compliance rate with Blue Book Standards guideline 1 is outlined in Table 7.

**Table 7:** Overall compliance rate with Blue Book Standards guideline 1

Compliance Rates	
Compliance rate pre-intervention	17.00%
Compliance rate postintervention	21.70%
Total compliance rate	18.30%

## Discussion

Acute hip fracture patients represent an increasingly complex and challenging cohort of patients to manage due to frailty, decrease in cognition and multiple medical comorbidities. The challenge of managing these patients is further compounded by overcrowded, understaffed, and under-resourced EDs. Patients frequently spend prolonged durations of time in the ED after formal admission has taken place but before transfer to an orthopedic trauma ward is possible, and this may be compounded by a delay in completing investigations [8].

As part of our analysis, we wanted to establish the duration of different stages along the patient journey through the ED to identify areas where the delivery of care could be optimized in order to meet the four-hour admission window. In our cohort, as a whole, the mean duration from arrival in ED to a diagnostic pelvic X-ray being requested was 59 minutes. On average, this diagnostic X-ray was not filmed and available for interpretation until two hours and eight minutes after the arrival of patients in the ED. This represents a major delay in the delivery of timely care to these patients, although it is worth noting that in the postintervention group, there was an improvement of 53 minutes in the time to completed X-ray with a statistically significant improvement in time to X-ray ordering (p=0.007). This key step represents the first area where there is room for significant improvement and optimization of care for these patients, as patients cannot progress to admission and ward transfer without a definitive diagnosis.

It has also been demonstrated in the cohort that the average time for blood to be drawn from a patient prior to arrival is 98 minutes. While this is not as significant a step in terms of advancing care for these patients as a pelvic X-Ray, this also represents a barrier to admission and compliance with BOA guideline 1 in many patients, as routine blood tests provide important information regarding concomitant acute medical issues, such as cardiac events, anemia, and high international normalized ratio (INR), which may need to be addressed emergently prior to ward transfer [9-10].

It is worth noting that the time of orthopedic referral improved significantly in the pre- and postintervention groups by 58 minutes on average. This was likely driven by increased awareness and drive by the ED to refer these patients so as to improve compliance with guideline one and meet hospital targets. However, it is worth noting that while the documented timings of these referrals have improved, 12.5% of these referrals are without a diagnostic X-ray and 25% are without blood being taken. At night, this increases to 16% of patients being referred without an X-ray and 60.8% of patients without blood tests. Given, the need for basic investigations to be completed in order to diagnose a potential hip fracture and other acute medical issues, this improvement in referral time does not necessarily expedite care, unless it occurs concurrently with the necessary pre-workup investigations.

Although the initial audit suggested the majority of gains could be achieved through improvements pre-referral, it also highlighted areas for potential improvement at all stages along the journey. Subsequently, a policy change was implemented, stipulating that on-call orthopedic clinicians would review prospective hip fracture patients prior to definitive diagnosis once the hip fracture alert was initiated. It has been shown in one clinical trial that paramedics can diagnose hip fractures out of the hospital, with 77% sensitivity [11]. The reliability of paramedic diagnosis is further demonstrated by the successful implementation of the bypass system in the South-East of Ireland where suspected hip fracture patients are triaged at the scene and brought directly to an orthopedic trauma center, bypassing potentially closer EDs in which orthopedic services are not directly available on site [12]. Therefore, it was felt that a pre-emptive orthopedic review of these patients was a reasonable intervention and may potentially expedite time to ward. As our center operates a central on-call rota where general surgery SHOs are frequently on call for orthopedics, uptake for this policy was mixed, although there was a seven-minute improvement in response times. When an orthopedic SHO was on call, we found that the time to review was substantially quicker although this did not significantly alter the time to ward transfers. Subsequently, while our intervention did demonstrate improvement and has the potential to play a role in expediting ward transfer in these patients, it is not sufficient alone to produce significant meaningful changes in time to ward transfer.

Based on these observations, it is clear that there are a number of challenges that need to be overcome in order to improve compliance with BOA guideline 1, noticeably, the commencement of an integrated hip fracture care pathway. Pollmann et al. describe a system in a similar institution to ours and have demonstrated ward transfer times of 1.1 hours and time to surgery of 23 hours in patients via a standardized fast track pathway through the ED to the ward [13]. Larsson et al. describe a system where the patients' initial diagnostic X-ray is expedited based on paramedic assessment, and this has been shown to statistically reduce time to ward transfer [14]. Fast-track pathways have been utilized for many years in the UK for hip fractures, with significant reductions in time to ward transfer [15-17]. A potential solution in our system is to utilize the generic hip fracture alert system but to extend the system to include an alert to both radiographers and porters when such patients present to the ED. This would ensure that these patients can be identified and brought to X-ray immediately on arrival to the ED where they can undergo pelvic and chest X-rays in an expedited fashion. Once the X-ray is filmed, this can be reviewed by the ED doctors and if a hip fracture is diagnosed, a standardized proforma can be initiated where blood tests, electrocardiograms (ECGs), urinary catheters, and multimodal analgesia can then be initiated by the ED in conjunction with an early orthopedic review. As the porters and orthopedic trauma ward have been alerted already, the patient can then be brought promptly to the ward, which would likely make a significant difference in the time to ward transfer. A change such as this would require a considerable culture shift within the organization, as there are multiple stakeholders across multiple departments that would need to be involved; however, it is hoped that data-driven studies such as this can help establish an integrated care pathway that can expedite care.

One of the other issues that have been highlighted as part of this analysis is the issue of documentation. The arrival of time to ward was documented in just 70.5% of cases overall, meaning that in almost a third of patients, compliance with BOA guideline 1 cannot be assessed. This has significant implications not only in terms of assessing performance but also in terms of payment of the BPT.

There are a number of limitations to our study. First, there is a small sample size, as we are assessing a single institutions' performance over a limited time frame. Given this small sample size, a large effect would have to occur in order to demonstrate a statistically significant difference between the pre- and postintervention groups. This could possibly explain the lack of statistical significance for many of our timestamps in the pre and postintervention groups. As well as this, despite collecting data over a similar timeframe, there are more patients in the pre-intervention group than the postintervention group. This is possibly reflective of the time of year the analysis occurs, with a higher rate of trauma occurring in the summer months relative to the winter [18]. There are a number of endpoints that are not assessed as part of our analysis. The timing of ECGs, other hematological investigations, and chest X-rays were not measured as part of our analysis and the absence or non-completion of these investigations may potentially delay transfer to ward in certain patients. Finally, as our intervention is reliant on the on-call team assessing the patients prior to referral, it is difficult to definitely assess how frequently this occurred, particularly in the absence of documented timestamps for hip fracture alert, ED referral, and orthopedic review in a number of patients.

## Conclusions

The transfer of hip fracture patients from the emergency department to the orthopedic trauma ward is a complex process that requires many independent events to happen promptly. Orthopedic response times are an important part of this process, however, for meaningful improvements in time to ward transfer to occur, a standardized care pathway with all clinical stakeholders involved needs to be initiated and adhered to.

## Appendices

**Table 8:** British Orthopaedic Association and British Geriatric Association Blue Book Standards for acute hip fracture care

Number	Guideline
1	All patients with hip fractures should be admitted to an acute orthopedic ward within 4 hours (240 minutes) of presentation.
2	All patients who are medically fit should have surgery within 48 hours of admission and within normal working hours.
3	All patients with hip fractures should be assessed and cared for with a view to minimizing their risk of developing a pressure ulcer.
4	All patients presenting with a fragility fracture should be managed on an orthopedic ward with routine access to acute orthogeriatric medical support from time of admission.
5	All patients presenting with fragility fractures should be assessed to determine their need for antiresorptive therapy to prevent future osteoporotic fractures.
6	All patients presenting with a fragility fracture following a fall should be offered multidisciplinary assessment and intervention to prevent future falls.
